# Rational Drug Design for *Pseudomonas aeruginosa* PqsA Enzyme: An *in silico* Guided Study to Block Biofilm Formation

**DOI:** 10.3389/fmolb.2020.577316

**Published:** 2020-10-15

**Authors:** Bilal Shaker, Sajjad Ahmad, Thi Duc Thai, Seong-il Eyun, Dokyun Na

**Affiliations:** ^1^84 Heukseok-ro, Dongjak-gu, Department of Biomedical Engineering, Chung-Ang University, Seoul, South Korea; ^2^National Centre for Bioinformatics, Quaid-i-Azam University, Islamabad, Pakistan; ^3^84 Heukseok-ro, Dongjak-gu, Department of Life Science, Chung-Ang University, Seoul, South Korea

**Keywords:** multidrug resistance, biofilm, quorum sensing, drug discovery, structure-based virtual screening

## Abstract

*Pseudomonas aeruginosa* is an opportunistic gram-negative bacterium implicated in acute and chronic nosocomial infections and a leading cause of patient mortality. Such infections occur owing to biofilm formation that confers multidrug resistance and enhanced pathogenesis to the bacterium. In this study, we used a rational drug design strategy to inhibit the quorum signaling system of *P. aeruginosa* by designing potent inhibitory lead molecules against anthranilate-CoA ligase enzyme encoded by the *pqsA* gene. This enzyme produces autoinducers for cell-to-cell communication, which result in biofilm formation, and thus plays a pivotal role in the virulence of *P. aeruginosa*. A library of potential drug molecules was prepared by performing ligand-based screening using an available set of enzyme inhibitors. Subsequently, structure-based virtual screening was performed to identify compounds showing the best binding conformation with the target enzyme and forming a stable complex. The two hit compounds interact with the binding site of the enzyme through multiple short-range hydrophilic and hydrophobic interactions. Molecular dynamic simulation and MM-PBSA/GBSA results to calculate the affinity and stability of the hit compounds with the PqsA enzyme further confirmed their strong interactions. The hit compounds might be useful in tackling the resistant phenotypes of this pathogen.

## Introduction

*Pseudomonas aeruginosa* is a gram-negative opportunistic bacterial pathogen that poses a significant threat to patients in hospital environments (Page and Heim, 2009). The World Health Organization (WHO) classified *P. aeruginosa* as the highest priority pathogen and declared an urgent demand for new antibiotics (Shrivastava et al., 2018). *P. aeruginosa* is extremely resistant to antibiotics due to intrinsic, evolved, and acquired mechanisms, such as decreased cell permeability, enzymatic drug inactivation via horizontal gene transfer, and biofilm development (Bonomo and Szabo, 2006; Zavascki et al., 2010). Moreover, the β*-*lactamase gene present in its genome makes cephalosporins and penicillin ineffective (Strateva and Yordanov, 2009). It has been estimated that biofilm-forming *P. aeruginosa* is responsible for 65% of patient mortality and antibiotic resistance (Strateva and Yordanov, 2009).

Biofilm-embedded bacteria are 1,000 times more resistant to antibiotics than planktonic bacteria, and the biofilm also enables the bacteria to avoid a host’s immune system (Gaddy and Actis, 2009). In a biofilm, bacterial cells are embedded inside the matrix of extracellular polymeric substances (EPSs) composed of proteins, exopolysaccharides, DNA, macromolecules, and lipids (Flemming and Wingender, 2001). All of these render the antibiotics impermeable and ineffective. The presence of persistent cells in biofilms also significantly contributes to their multidrug resistance property (Lewis, 2007). Overall, the complex morphology of biofilms is a significant barrier in designing potent therapeutic agents that can eradicate biofilm-associated infections successfully.

Quorum sensing (QS), a cell-to-cell communication system, reportedly plays a pivotal role in establishing persistent infections (Hentzer et al., 2003; Alhede et al., 2009; Van Gennip et al., 2009; Chiang et al., 2013). QS relies on the processing, secreting, and sensing of small diffusible quorum sensing signal molecules (QSSMs), known as autoinducers (Waters and Bassler, 2005). When a bacterial community reaches a certain threshold (density), which is reflected by AI concentration in the surrounding environment, bacterial gene transcription in the community becomes synchronized, which enables the community to behave collectively. A wide range of activities is controlled by AIs, including virulence factor secretion, swimming motility, secondary metabolite production, biofilm maturation, and antibiotic resistance (Ng and Bassler, 2009). A promising approach to control the growth of this pathogen is to interfere with QS-mediated signaling, which disrupts bacterial communication and attenuates virulence so that the bacteria can be eliminated from the host (Mühlen and Dersch, 2016; Wagner et al., 2016; Kamal et al., 2017). Therefore, QS inhibitors can be very useful and effective as a prophylactic to control antibiotic-resistant bacteria.

**FIGURE 1:**
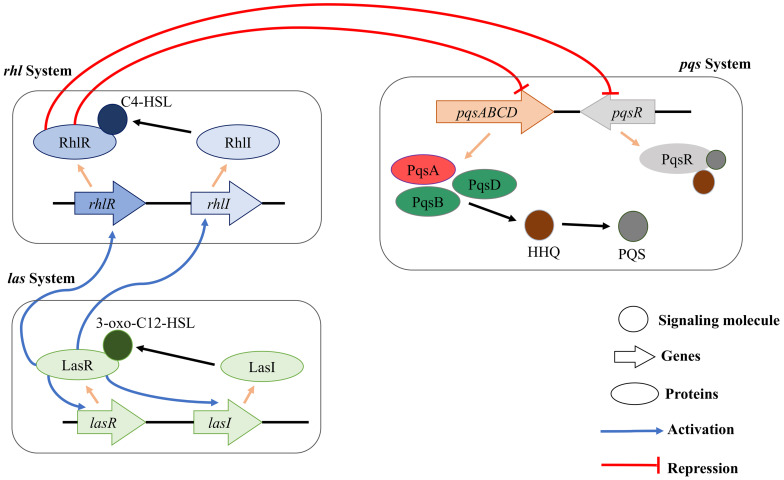
Schematic diagram of rhl, pqs, and las QS systems in *P. aeruginosa.* The three major QS systems use C4-HSL, 3-oxo-C12-HSL, and HHQ/PQS for cell-to-cell communication. In addition, the three QS systems can interact with each other for facilitating a complex regulation of the physiology of *P. aeruginosa*.

*Pseudomonas aeruginosa* has three major QS systems (*rhl, las*, and *pqs*) that mediate cell-to-cell communication and control the synthesis and secretion of virulence factors (Williams and Cámara, 2009; Jimenez et al., 2012; Figure 1). For instance, the *las* system positively regulates both the *rhl* and *pqs* systems by initiating the expression of AI receptors (RhlR and PqsR). RhlR and PqsR are also transcriptional activators when their respective AIs are bound. The *las* and *rhl* systems use two different AIs (acyl-homoserine-lactones, AHLs) (Alhede et al., 2009). The *pqs* system employs two signal molecules: 2-heptyl-3-hydroxy-4(1H)-quinoline or *Pseudomonas* quinolone signal (PQS) (Pesci et al., 1999) and its biosynthetic precursor 2-heptyl-4-hydroxyquinoline (HHQ) (Xiao et al., 2006). When PQS or HHQ molecules are bound to the PqsR transcriptional activator, PqsR induces the expression of various virulence genes, their biosynthetic genes, and biofilm formation-related genes (Calfee et al., 2001; Cao et al., 2001; Xiao et al., 2006). Although both PQS and HHQ bind to and activate PqsR, PQS is 100-fold more potent than HHQ (Xiao et al., 2006). Because the enzyme (PqsA) is responsible for the synthesis of the PQS signal molecule (Grandclément et al., 2016), inhibition of the PqsA enzyme can disrupt biosynthesis of PQS signal molecule and consequently PqsR-dependent gene regulation and ultimately biofilm formation. The PQS act as a linker of the *las* and *rhl* quorum sensing systems in *Pseudomonas aeruginosa* and it has been shown that PQS system showed activity even in the *lasR* mutant strains, which represents that PQS could be a more significant modulator in the quorum sensing (McKnight et al., 2000). Also, PQS system is a broad regulatory system in *P. aeruginosa* influencing iron acquisition, outer membrane vesicle production, cytotoxicity through oxidative stress, and immune responses in the host cell (van Kessel, 2019).

To date, several SAR studies have been conducted to identify the potential inhibitors for QS system in *P. aeruginosa* and also provide important scaffolds for future QS inhibitor development (Ji et al., 2016; Fong et al., 2017). According to reported studies, sulfur-containing compounds possess excellent QS inhibiting properties and can demolish the bacteria virulence.

In this study, we predicted putative QS inhibitor based on *in silico* drug discovery technology. We prepared a library of 521 compounds containing dialogs of reported inhibitors. Further, their binding affinities were predicted through molecular docking to screen the most potent binder. Subsequently, Molecular Dynamic (MD) simulation was performed to validate the screened putative QS inhibitors affinity for the docked site and understand the dynamic behavior of the enzyme. Finally binding free energy for hit drug compounds was calculated with the MM-PBSA/GBSA approach (Miller et al., 2012), which leads to a possible mechanistic calculation useful for future experimental analysis. Our *in silico* results support the prediction that these predicted compounds can efficiently suppress the PqsA QS system and block virulence.

## Materials and Methods

### Preparation of Enzyme Structure

To understand the interactions of the enzyme with ligands, the 3D structure of the target enzyme is required. Although the PqsA enzyme structure has not been experimentally proven, we subjected the protein sequence of *P. aeruginosa* PqsA (UniprotKB ID: Q9l4 × 3) to homology modeling using MODELLER9.24 (Eswar et al., 2007). In structural biology, comparative modeling or homology modeling is the most promising technology for significantly narrowing down the gap between experimentally determined structures and known protein sequences (Xiang, 2006). Two templates with maximum sequence identity and query coverage were used for better homology structure prediction (Template 1: PDB ID = 5OE3, query coverage = 77%, sequence identity = 100% and Template 2: PDB ID = 1ULT, query coverage = 96%, sequence identity = 25%). Multiple sequence alignment (MSA) of PqsA and the two templates was performed (Supplementary Figure S1). To date, only N terminal domain of the enzyme is determined due to the difficulty in getting the full-length protein structure. In this study, by using a close homology template we predicted the remaining structure of the PqsA in addition to the available N-terminal domain. It is significant to highlight the dynamic aspect of full-length protein upon ligand or drug molecules binding (Itoh et al., 2015; Flydal et al., 2019). However, in this study the C-terminal region did not affect the binding pocket of the N-terminal domain, we used only the N-terminal domain structure for putative inhibitor screening. Different online servers were used for structure assessment, including the Swiss-Model structure assessment tool (Waterhouse et al., 2018), Ramachandran plot (Gopalakrishnan et al., 2007), and PDBsum (De Beer et al., 2014). University of California San Francisco (UCSF) Chimera (version 1.14) (Pettersen et al., 2004) was used for structural visualization and energy minimization of a total 1,500 steps divided into the first 750 steps of the steepest descent and the last 750 steps of the conjugate gradient (Ahmad et al., 2017). During minimization, standard residues were treated with AMBER ff14SB force-field.

### QS Inhibitor Preparation

The QS inhibitors of PqsA were selected from those reported across various studies in the literature (Ji et al., 2016; Fong et al., 2017) and used as positive controls in this study. Moreover, 521 structural analogs to the reported inhibitors were generated from online repositories, such as ChemSpider (Ayers, 2012), ChEMBL (Davies et al., 2015), and PubChem (Kim et al., 2019). These web-servers provide a search module where users can find the available structures having a similar chemical composition (similar scaffolds) of the query structure. The similarity index was set to >85%, compounds having more than 85% structural similarity were selected for analog library. Discovery Studio (DS) and UCSF Chimera were used for ligand preprocessing, including protonation; ionization; and addition of explicit counter ions, hydrogen atoms, or atomic partial charges. Energy minimization was performed using forcefield AMBER ff14SB for small molecules. The refined dataset was further utilized for computational experiments.

Here, the previously reported QS inhibitors of PqsA were used for conducting a docking study to reveal their binding affinity toward the target enzyme. ChemDraw (Mendelsohn, 2004), a chemical drawing tool, was used to draw 2D structures of the compounds and to convert the compounds into 3D structures. The compounds were eventually subjected to ligand preprocessing.

### Ligand Binding Domain Analysis

To date, only the functional N-terminal domain of PqsA has been analyzed (Witzgall et al., 2017). For a brief binding pocket analysis, Witzgall et al. compared the N-terminal binding domain of PqsA from *P. aeruginosa* with the other CoA ligase-like anthraniloyl-CoA transacylase AuaE from *Stigmatella aurantiaca* (Sandmann et al., 2007), the PqsA ortholog HmqA from *Burkholderia pseudomallei* and *B. ambifaria* (Diggle et al., 2006; Vial et al., 2008), and an anthranilate-CoA from *Azoarcus evansii* (Schühle et al., 2001). According to the reported study, PqsA possesses highly conserved residues when compared with other aryl-CoA ligases, such as Gly307, Gly302, Ala278, Gly279, His308, and Tyr211.

### Molecular Docking

Molecular docking was performed on the analog library through the PyRx interface (Dallakyan and Olson, 2015) of AutoDock Vina (Trott and Olson, 2010). During docking, the following parameters were applied: exhaustiveness was set to 300, residues of the N-terminal domain involved in the binding pocket were established as the binding site, and the maximum number of poses was set to 300. The PyRx tool generates binding affinity values in the negative (a larger negative value implies a stronger binding affinity. The top 300 inhibitors showing a high binding affinity were retrieved for further analysis. The docking results of the top 10 inhibitors are shown in Supplementary Table S1. The docking protocol used herein was first tested by docking a known co-crystallized compound at the N-terminal structure of PqsA (PDB ID = 5OE3) with 300 iterations. We found the same binding mode of the known inhibitors determined in crystallization studies. Docking reproducibility results by AutoDock Vina are shown in Supplementary Figure S2.

A brief binding analysis, including interaction, binding angles, binding poses, binding residues, and bond lengths, was visually conducted using DS, UCSF Chimera, and LigPlot (Wallace et al., 1995). Ligands with a strong binding affinity [6-amino-9-((2R,3R,4S,5R)-5-(((N-(2-aminobenzoyl)sulfamoyl)oxy)methyl)-3,4-dihydroxytetrahydro furan-2-yl)-3H-purine-1,7,9-triium—named compound 1066—and 6-amino-9-((2R,3R,4S,5R)-3,4-dihydroxy-5-(((N-(3-hydroxy-2-naphthoyl)sulfamoyl)oxy)methyl)tetrahydrofuran-2-yl)-3H-purine-1,7,9-triium—named compound 1084] to the active pocket were selected as more potent QS inhibitors of PqsA from *P. aeruginosa.*

### Bioavailability

The physicochemical properties of the molecule, including Lipinski’s rule of five (molecular weight <500 Dalton, H-bond donor <5, H-bond acceptor <10, and cLog*P* < 5), toxicity prediction (tumorigenic, mutagenic, and irritant), and drug-likeness, were predicted using SwissADME (Daina et al., 2017) and PreADME (Lee et al., 2004).

### Molecular Dynamic (MD) Simulations

The dynamic behavior of top docked complexes was studied using molecular dynamic (MD) simulations. The top two docked complexes were used in MD simulations carried out through the Sander Module of AMBER (Assisted model building with energy refinement) (Case et al., 2016). Primary coordinates of docked complexes were performed for MD simulations, including energy minimization (for the complete system, water and heavy atoms of the system), heating (at 300 K for the 20 picoseconds), equilibration (for 100 picoseconds with a time step of 2 nanoseconds), pressure (for 50 picoseconds), and production (for 100 ns). A general amber force field (GAFF) (Dickson et al., 2012) was used for putative inhibitor while ff03.rl (Case et al., 2014) used for the enzyme. Sodium ions were added randomly to neutralize the system. A simulation production run of 50 ns was accomplished to evaluate the dynamics of the complex and check the docked conformation stability of the ligand. Langevin dynamics (Izaguirre et al., 2001) were used for temperature and pressure control, whereas the SHAKE algorithm (Kräutler et al., 2001) was applied for correct bond length. The production run was performed in constant volume and temperature (NVT) ensemble (Nosé, 1984) using the Berendsen algorithm (Lemak and Balabaev, 1994). MD simulation trajectories of each nanosecond were recorded and visualized and analyzed with Visual Molecular Dynamic (VMD) (Humphrey et al., 1996).

### Binding Free Energies of Complexes

The binding free energy for top docked complexes was calculated using two methods: Molecular Mechanics Poisson-Boltzmann Surface Area (MMPBSA) and Molecular Mechanics-Generalized Born Surface Area (MMGBSA) incorporated with MMPBSA.py module of AMBER18 (Miller et al., 2012). In total, 50 frames from the trajectories were processed and the net energy of the system was calculated through the following equation.

ΔG=B⁢i⁢n⁢d⁢i⁢n⁢gΔG-C⁢o⁢m⁢p⁢l⁢e⁢xΔG-R⁢e⁢c⁢e⁢p⁢t⁢o⁢rΔGI⁢n⁢h⁢i⁢b⁢i⁢t⁢o⁢r

Each of the terms in the equation involves the calculation of several energy components, including van der Waals energy, electrostatic energy, and internal energy summed from molecular mechanics and polar contribution toward solvation energy. The analysis also takes into account the contribution from non-polar terms toward solvation energy and inhibitor entropy.

## Results and Discussion

### Overall Structure Assessment of PqsA

In rational drug discovery, the most fundamental step is to obtain a 3D structure of the target protein. The 3D structure is used to understand the structural details and molecular function and to discover potent inhibitors of the target enzyme (Kopec et al., 2005). Because the full 3D structure of the PqsA from *P. aeruginosa* was unavailable, we performed comparative modeling using 5OE3 (PqsA N-terminal domain) from *P. aeruginosa* PAO1 and 1ULT from *Thermus thermophilus* using MODELLER9.24 (Eswar et al., 2007).

The predicted PqsA 3D structure is composed of a large N-terminal domain and a small C-terminal domain, and the domains are connected by a small flexible hinge (Gulick, 2009; Supplementary Figure S3). The N-terminal domain of PqsA can be subdivided into three subdomains: two β-sheets connected by an internal 2-fold symmetry surrounded by α-helices and a distorted β-sheet followed by a flexible hinge region that links the N-terminal and C-terminal domains.

**FIGURE 2:**
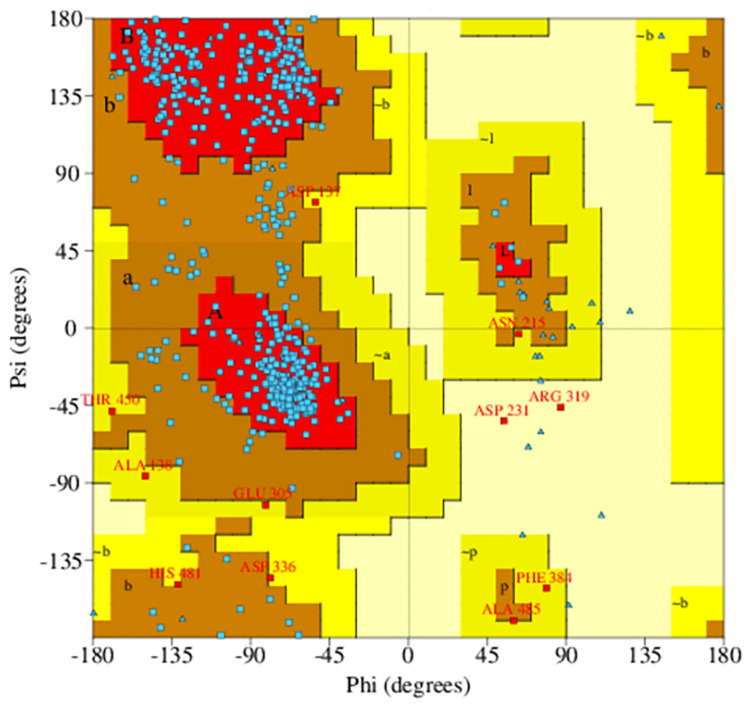
Ramachandran plot of predicted PqsA structure. The blue dots indicate torsion angles distributed along the core secondary structure regions of the enzyme (shown in red). Yellow and pale-yellow areas contain favored and generously allowed torsion angles, respectively.

Ramachandran plot analysis was performed to assess the quality of the predicted structure. Most residues (77.5%) of the structure were grouped in the most favored region, whereas only 0.5% of the residues lie in the outlier region, which indicates a good structure quality (Figure 2). The secondary structure contained the following: 17.6% strand, 20.3% alpha helix, 2.1% 3–10 helix, and 60% gamma turns, beta turns, helix-helix interactions, beta bulges, beta hairpins, and beta alpha beta motifs. The 2D structure of the protein was also predicted using MODELLER software, and it is illustrated in Supplementary Figure S4.

### Reported QS Inhibitors of PqsA

**FIGURE 3:**
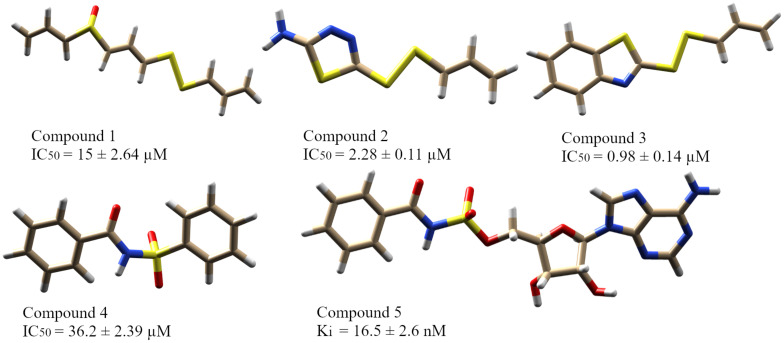
The five reported PqsA inhibitors. The compounds are displayed in a stick representation along with their respective inhibitory constant values.

In this study, five recently reported PqsA inhibitors were retrieved from the literature (Ji et al., 2016; Fong et al., 2017) and used as reference compounds to discover more potent inhibitors (Figure 3). The inhibiting potency of these compounds has been experimentally measured (Ji et al., 2016; Fong et al., 2017). The IC_50_ values of compounds 1–4 were 15 ± 2.64, 2.28 ± 0.11, 0.98 ± 0.14, and 36.2 ± 2.39 μM, respectively. For compound 5, its constant K_*i*_ used to represent inhibitory potency was 16.5 ± 2.6 nM. Structure comparison of known compounds determined that compound 4 and 5 have a significant structure similarity, the 3D structures of the five compounds are shown in Figure 3.

A library of 521 analogous compounds to the reported PqsA inhibitors was generated to screen for potent inhibitory compounds of PqsA by utilizing similarity search tools from online compound databases (PubChem, ChemSpider, and ChEMBL) based on 85% structural similarity to the known selected inhibitors.

After a brief docking analysis, including binding affinity, binding poses, interacting residues, and enzyme–inhibitor interactions of all the compounds (five reported PqsA inhibitors and the analog library), compounds with a stronger binding affinity than that of controls were selected as potent PqsA inhibitory compounds.

### Molecular Docking

Protein-ligand docking plays a pivotal role in predicting the accurate orientation of a ligand with its target protein (Morris and Lim-Wilby, 2008). For better understanding, molecular docking was divided into two phases: (1) enzyme docking with the known PqsA inhibitors and (2) enzyme docking with the analog library. Compounds with a strong binding affinity and correct binding poses were selected. Binding affinity denotes the sum of total torsional energy, internal energy, and intermolecular energy subtracted from the unbound energy system. A strong binding affinity conformation indicates a stable protein-ligand complex. We also found that the compounds docked in similar conformations to both full-length PqsA structure as well as to the N-terminal structure only. As no significant contribution of the C-terminal on compounds binding at the N terminal was found, this study proceeded with the N-terminal structure only.

### Enzyme Docking With Reported QS Inhibitors

**FIGURE 4:**
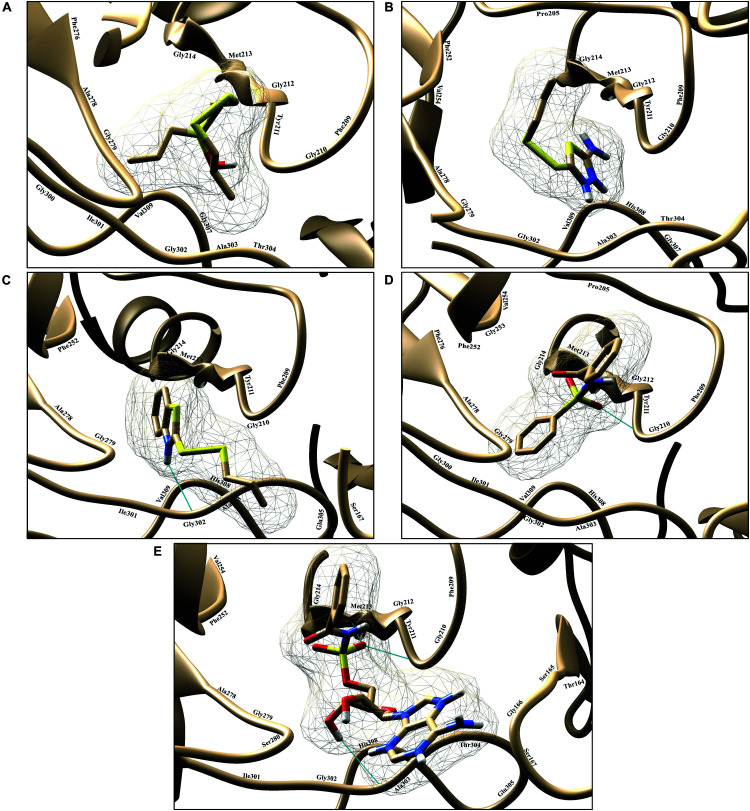
Illustration of PqsA binding with five known inhibitors. The five known inhibitors are rendered in the active pocket: **(A–E)** correspond to compound 1–5. Inhibitors are displayed in stick representation, and backbone hydrogen bonds are shown as cyan-colored lines.

To find the binding poses, interactions, and binding affinity of all the five known inhibitors, they were docked into the binding pocket of PqsA using the PyRx tool. Consequently, 300 poses for each inhibitor were generated, and inhibitors with a strong binding affinity were selected for further analysis (Figure 4). The docking results showed that the binding affinity ranged from −4.4 to −8.5 kcal/mol. Docking analysis exhibited that the shared motif in compound 4 and 5 binds to the same residues (Val254, Phe209, Pro205, Phe252, Ala278, Tyr211, Gly279, Gly214, and Ile310), and the identical hydrogen bond of the shared motif in compound 4 and 5 was observed with the active site residues (Tyr211 and Gly214). There is a correlation between IC_50_ values of compounds and binding affinities of known inhibitors, except compound 4 that has a low IC_50_ value but high binding affinity. One possible reason for this could be that compound 4 has a large structure and more interactions, which produced a higher binding affinity, considers as a limitation of binding affinity-based inhibitor design. The interactions between each known inhibitor and PqsA are depicted in Supplementary Figure S5.

### Docking Analysis of Top Hits From Analog Library

**FIGURE 5:**
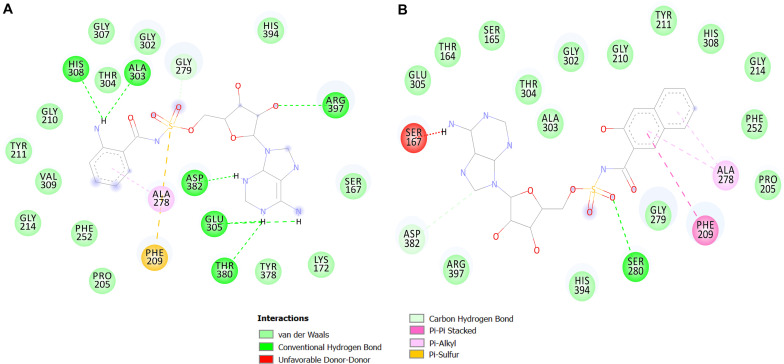
2D interaction maps of the top two predicted compounds. The compounds 1066 **(A)** and 1084 **(B)** are shown.

The library containing 521 analog compounds was used for virtual screening to find more potent compounds to inhibit the QS system in *P. aeruginosa.* A brief binding analysis was performed for the top complexes with a high binding affinity to select the potent *pqs* QS-binder. As a result, the top two hit compounds were selected from the library, and these compounds were predicted to have greater potential than the previously reported compounds. Both top compounds (compound 1066 and 1084) have a significant structural similarity and also their shared motif docked in a similar position by making interactions with conserved active residues within the binding pocket. 2D interactions of the top two hit compounds are depicted in Figure 5.

**FIGURE 6:**
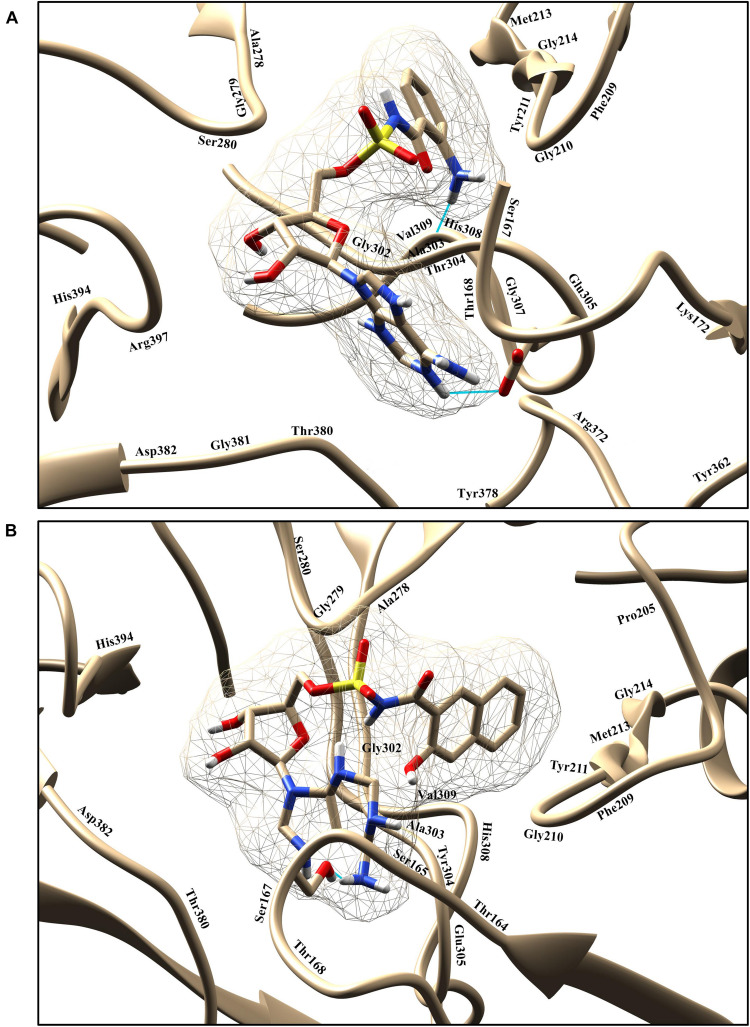
Graphical representation of binding poses and residues that interact with the compounds 1066 **(A)** and 1084 **(B)**. The hydrogen bond is represented in cyan color.

The top two predicted compounds showed a stronger binding affinity with the conserved residues in the binding pocket (Figure 6). Both compounds contain one sulfur group in their structures. Generally, compounds with sulfur scaffolds, including antibiotics, antifungal agents, antitumor agents, and enzyme inhibitors, are related to a wide range of bioactivities (Feng et al., 2016).

Compound 1066 possessed seven conventional hydrogen bonds with the key residues, His308, Ala303, Arg397, Asp382, Thr380, and two bonds with Glu305; one carbon-hydrogen bonds with Gly279; one pi-alkyl interaction with Ala278; and one pi-sulfur interaction with Phe209. The residues Tyr211, Gly210, Gly307, Thr304, Gly302, His394, Ser167, Lys172, Tyr378, Pro205, Phe252, Gly214, and Val309 took part in van der Waals interactions. The binding affinity of the compound 1066 with PqsA was −9.1 kcal/mol.

Compound 1084 also showed a very strong binding affinity (^_^9.3 kcal/mol) with PqsA by forming one conventional hydrogen bond with Ser280; one carbon-hydrogen bond with Asp382; one pi–pi stacked interaction with Phe209; and two pi-alkyl bonds with Ala278. The residues Glu305, Thr167, Ser165, Thr304, Ala303, Gly302, Gly210, Tyr211, His308, Gly214, Phe252, Pro205, Gly279, His394, and Arg397 were involved in van der Waals interactions with the compound, while one residue (Ser167) is involved in unfavorable donor-donor interaction.

The two hit compounds are analogs of compound 5 and share a significant portion of the structure. Compound 5 has been experimentally proved as a potent QS inhibitor exhibiting a Ki value of 16.5 ± 2.6 nM and a predicted binding affinity of −8.5 kcal/mol. Compared with the compound 5, the two hit compounds showed higher binding affinities of −9.3 kcal/mol and −9.1 kcal/mol, which represents that the two hits could be a high-affinity binder of PqsA enzyme. The hit compounds share a structural motif. The motifs of the hit compounds interact with the conserved active site residues in the same docking pose. RMSDs of the docked complexes with the two hits were 0.000Å, which indicates that there is no significant variability in enzyme structure and ligand binding pose.

**FIGURE 7:**
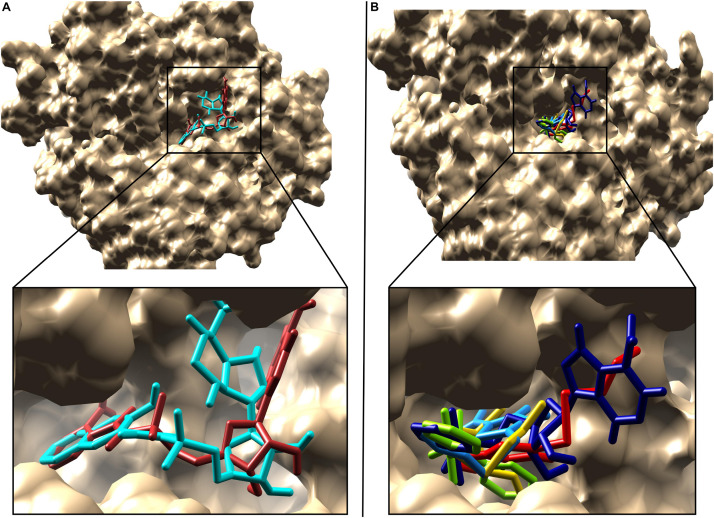
Superimposition of the reported and predicted PqsA inhibitors. Inhibitors are displayed in stick representation, **(A)** superimposition of the top two predicted PqsA binders, compound 1066 is in brown while compound 1084 in cyan color and **(B)** Superimposition of the five reported PqsA inhibitors within the binding pocket. Yellow, blue, red, chartreuse and navy-blue colors represent compounds 1–5, respectively..

In the docking analysis with known inhibitors, the residues (Pro205, Phe252, Met213, Gly214, Phe209, Ala278, Gly279, Tyr211, Gly210, His308, Gly302, and Ala303) were found to be involved in the interactions. We also found that Tyr211, Pro205, Phe252, Gly214, Phe209, Ala278, Gly279, Gly210, His308, Gly302, and Ala303 residues are also involved in the interactions with the compounds 1066 and 1084. Interestingly, Ala278 that is involved in the interactions of all inhibitors and is also involved in the interactions of compounds 1066 and 1084. This represents that the two hit compounds interact with the binding pocket in a similar manner with known inhibitors. This result provides important information in designing PqsA inhibitors. A brief graphical docked complex representation of both reported and predicted compounds has been depicted in Figure 7 and their binding affinities are shown in Table 1.

**TABLE 1:** Binding affinity and interacting residues of the five reported compounds and two predicted analogs.

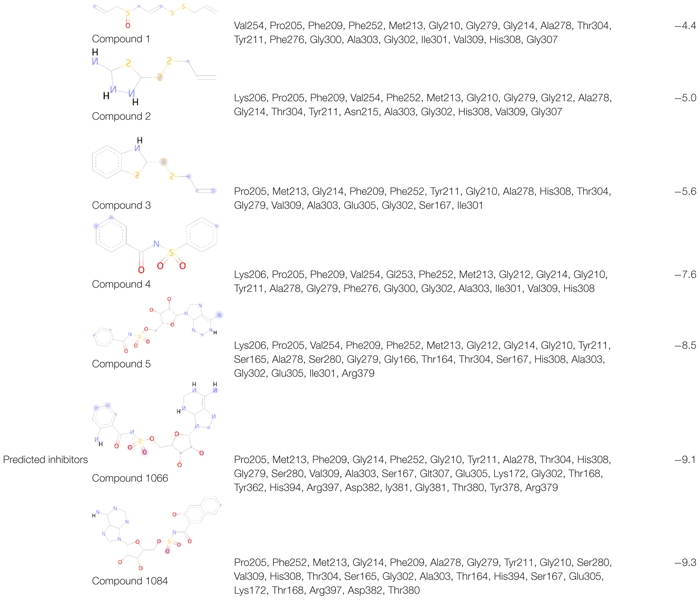

### Molecular Dynamics Simulation

**FIGURE 8:**
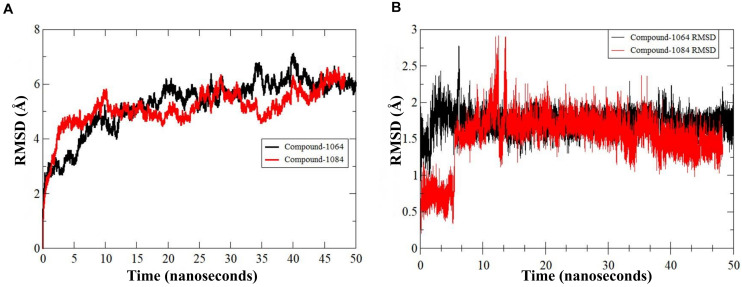
RMSD of PqsA protein in the presence of a compound. **(A)** Structure dynamics RMSD of PqsA protein in the presence of ligand **(B)** ligand dynamics RMSD in the PqsA protein pocket.

**FIGURE 9:**
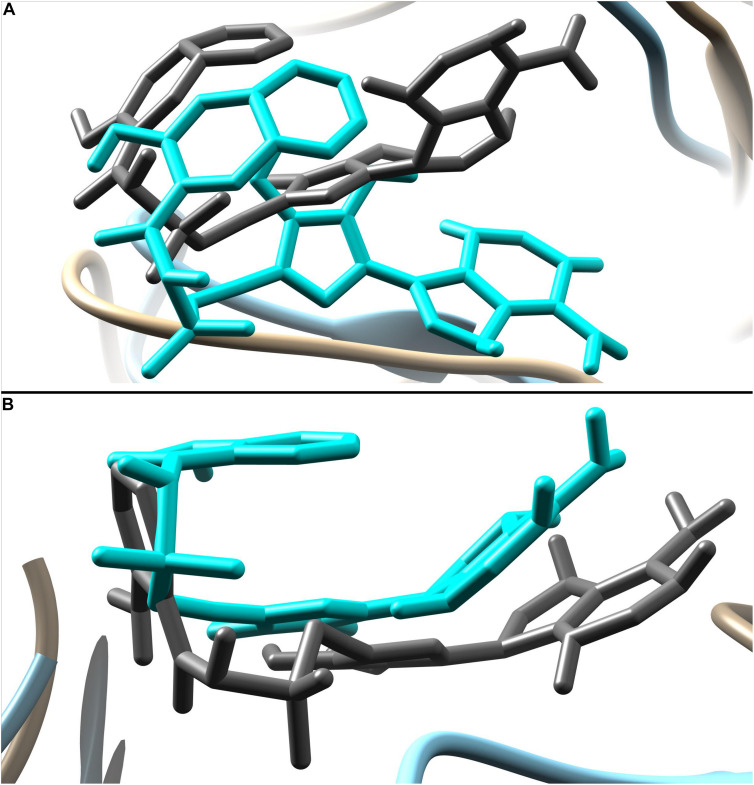
Ligand movement from 1 to 50 ns. Ligand pose at 1 ns is represented by dim gray color while pose at 50 ns is in cyan. **(A)** Movement of compound 1084 and **(B)** movement of compound 1066.

The predicted two complexes comprised of a chemical compound and PqsA enzyme were used in molecular dynamics simulation to unveil the dynamics of the enzyme in the presence of a ligand as well as to confirm the conformation stability of ligands predicted by the docking simulation. For the two compounds, the initial conformations docked on the PqsA were stable and no major changes were observed in terms of ligand RMSD (Figure 8). The mean RMSD of compound 1064 was 1.7 Å, and the mean RMSD of compound 1064 was 1.51 Å. This result confirmed that the predicted binding modes by docking are stable and consistent. Ligand movements during the simulation from 1 to 50 ns are depicted in Figure 9.

### MM-PBSA/GBSA Binding Free Energy Analysis

MM-PBSA method has been recently used to estimate the binding free energy of a given complex in rational drug discovery. As shown in Table 2, the gas phase energies (ΔG_*gas*_) in both complexes are very high mainly due to the significant contribution of electrostatic energy (compound 1064) and the decent role of van der Waals energy (compound 1084). In contrast, the net solvation energy (ΔG_*solv*_) is less favorable in both systems. Nevertheless, the total binding free energies of both complexes are promising: PqsA-compound 1064 has −18.4 kcal/mol in MM-GBSA and −31.4 kcal/mol in MM-PBSA, and PqsA-compound 1084 has −27.4 kcal/mol in MM-GBSA and −34.4 kcal/mol in MM-PBSA. These values reflect the stable complex conformation and high intermolecular affinity.

**TABLE 2:** Binding Free Energies of the complexes.

	**MMGBSA**				**MMPBSA**			

	**Energy Component**	**Average**	**Std. Dev.**	**Std. Err. of Mean**	**Energy Component**	**Average**	**Std. Dev.**	**Std. Err. of Mean**
Compound 1064	VDWAALS	–35.1814	3.2543	0.3254	VDWAALS	–35.1814	3.2543	0.3254
	EEL	–196.9409	10.4290	1.0429	EEL	–196.9409	10.4290	1.0429
	EGB	218.0214	8.8511	0.8851	EPB	203.9634	8.6698	0.8670
	ESURF	–4.3052	0.1455	0.0146	ENPOLAR	–3.2338	0.0879	0.0088
	−	−	−	−	EDISPER	0.0000	0.0000	0.0000
	ΔG gas	–232.1223	9.5819	0.9582	ΔG gas	–232.1223	9.5819	0.9582
	ΔG solv	213.7161	8.8466	0.8847	ΔG solv	200.7296	8.6597	0.8660
	ΔTOTAL	–18.4062	3.2335	0.3233	Δ TOTAL	–31.3927	3.3925	0.3392
Compound 1084	VDWAALS	–57.6556	2.7124	0.2712	VDWAALS	–57.6556	2.7124	0.2712
	EEL	17.7792	8.4691	0.8469	EEL	17.7792	8.4691	0.8469
	EGB	17.4195	7.8428	0.7843	EPB	9.6471	10.1448	1.0145
	ESURF	–4.9013	0.1551	0.0155	ENPOLAR	–4.1481	0.0759	0.0076
	−	−	−	−	EDISPER	0.0000	0.0000	0.0000
	ΔG gas	–39.8764	9.5508	0.9551	ΔG gas	–39.8764	9.5508	0.9551
	ΔG solv	12.5181	7.8326	0.7833	ΔG solv	5.4990	10.1226	1.0123
	ΔTOTAL	–27.3583	3.5233	0.3523	Δ TOTAL	–34.3774	5.1235	0.5124

### Bioavailability

The bioavailability of the two selected compounds is important in drug discovery because it determines the applicability of the compounds as a drug. Pharmacophoric properties, including Lipinski’s rule of five and toxicological properties, are noteworthy to determine drug accessibility. Ideal drug candidates exhibit the following pharmacophoric properties. Drugs should satisfy Lipinski’s rule of five (molecular weight < 500 g/mol, H-bond donor < 5, H-bond acceptor < 10, and cLog*P* < 5). The topological polar surface area (TPSA) should range from 20 to 130 Å. Lipophilicity should range from −0.7 to +6.0, and more negative values indicate lower skin permeability. Online servers (SwissADME and PreADME) were used to evaluate the physicochemical properties of the top two hit compounds (Table 3). Important physicochemical properties of the hit compounds were predicted, which included Lipinski’s rule of five, PAINS assay, lipophilicity, TPSA, and, more importantly, bioavailability. All these predicted physicochemical properties of the two compounds were suitable enough to attempt experimental evaluations. Therefore, these compounds could be new candidate agents for the efficacious management of bacterial infections.

**TABLE 3:** Physicochemical properties of hit compounds.

**Compound**	**MW (g/mol)**	**Lipinski’s rule of five**	**PAINS**	**Lipophilicity**	**TPSA (Å^2^)**	**Synthetic accessibility**	**Water solubility**	**Bioavailability score**	**BBB Permeation**	**Skin permeability (cm/s)**
Compound 1066	462.48	0 violations	0 alerts	0.07	187.51	4.69	Soluble	0.55	No	−8.91
Compound 1084	513	1 violation	0 alerts	1.33	181.72	4.84	Moderately soluble	0.17	No	−8.10

## Conclusion

Inhibition of the *P. aeruginosa* quinolone signaling system is an attractive and promising approach to impede infections by preventing biofilm formation. In this study, we present the applications of rational *in silico* drug discovery techniques to identify novel and more putative inhibitors for PqsA, an important enzyme in *P. aeruginosa* quinolone signaling. Based on virtual screening, we identified two compounds (compounds 1066 and 1084) as potent compounds showing a good affinity for the PqsA enzyme. Both these compounds have vital chemical moieties responsible for important chemical interactions with hotspot residues of the PqsA enzyme. The length of the compound also seems important because it provides a balanced network of chemical interactions at the docking site. The affinity and stability of the compounds binding mode were examined through molecular dynamic simulation and MMPB/GBSA assay both are in strong agreement of strong intermolecular affinity and formation of stable complexes. Furthermore, a suitable profile of drug-like properties and pharmacokinetics was revealed for both compounds, thereby increasing their chances of being good leads. Based on the findings, we believe that the compounds should be subjected to *in vitro* and *in vivo* investigations to affirm their potency and could be used in further structural optimization of new potent derivatives.

## Data Availability Statement

The original contributions presented in the study are included in the article/Supplementary Material, further inquiries can be directed to the corresponding author.

## Author Contributions

BS, SA, and DN: conceptualization. BS: data curation. BS and S-iE: methodology. DN: supervision. BS and DN: manuscript writing. All authors have read and agreed to the published version of the manuscript.

## Conflict of Interest

The authors declare that the research was conducted in the absence of any commercial or financial relationships that could be construed as a potential conflict of interest.
