# Supplementary material for: Rational Drug Design for Pseudomonas aeruginosa PqsA Enzyme: An in silico Guided Study to Block Biofilm Formation
Source: Front Mol Biosci. 2020 Oct 15;7:577316. doi: 10.3389/fmolb.2020.577316 (PMC7593710; doi:10.3389/fmolb.2020.577316)
Supplement: Supplementary file 1 [file Data_Sheet_1.DOCX]

Supplementary Materials

Rational drug design for *Pseudomonas aeruginosa* PqsA enzyme: An *in silico* guided study to block biofilm formation

Bilal Shaker, Sajjad Ahmad, Thi Thai Duc, and Dokyun Na


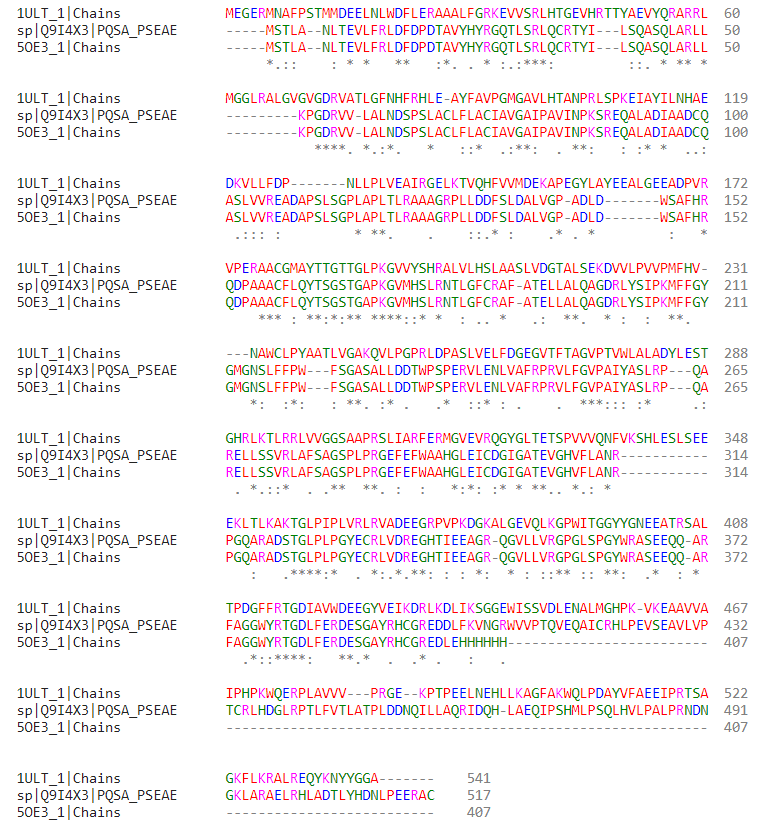


**Supplementary Fig. 1.** MSA between the target PqsA enzyme and used templates.


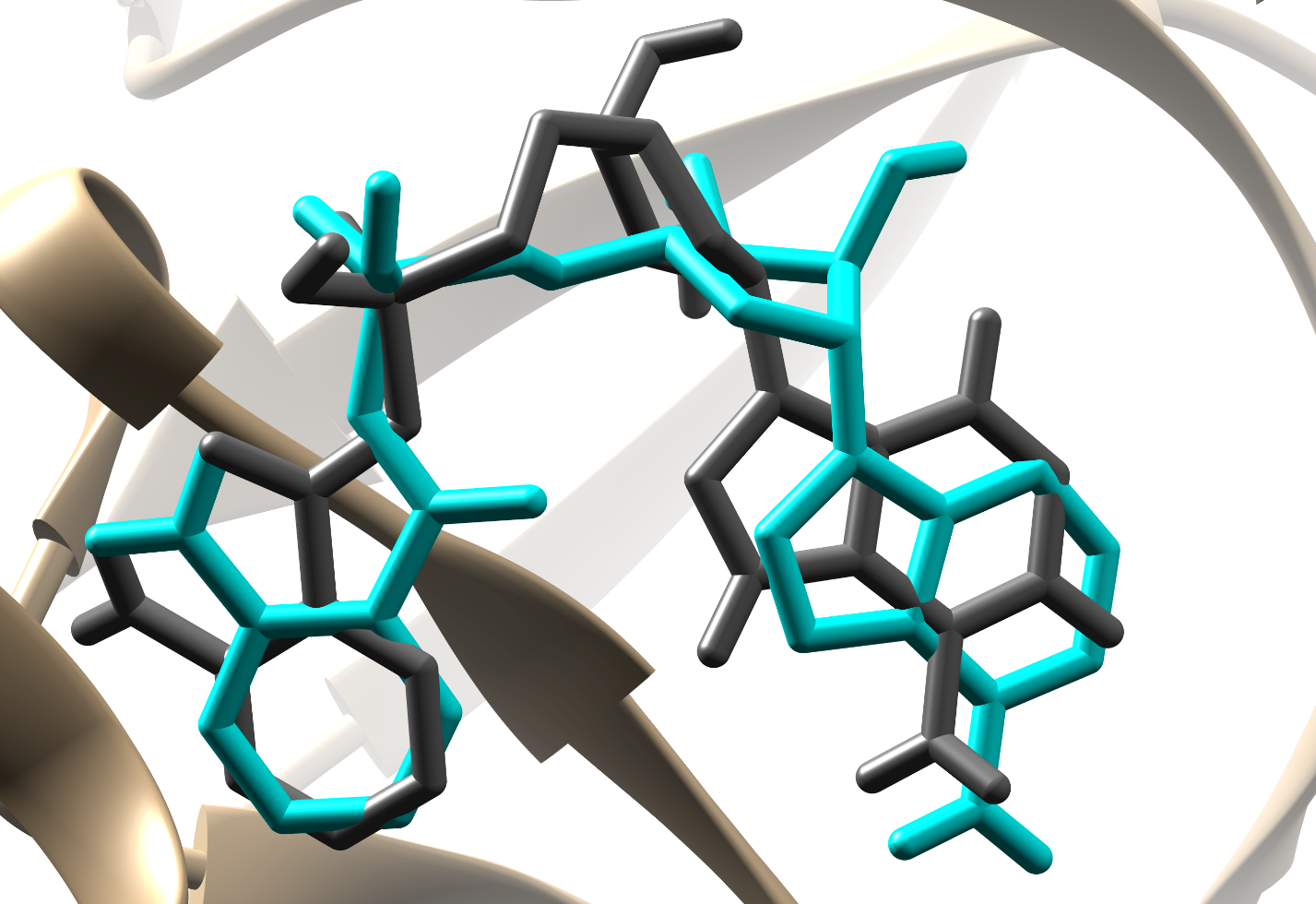


Supplementary Fig. 2. Docking pose reproducibility by using AutoDock Vina. The reported docking pose and the pose predicted by AutoDock Vina are represented in dim-gray and cyan color, respectively.


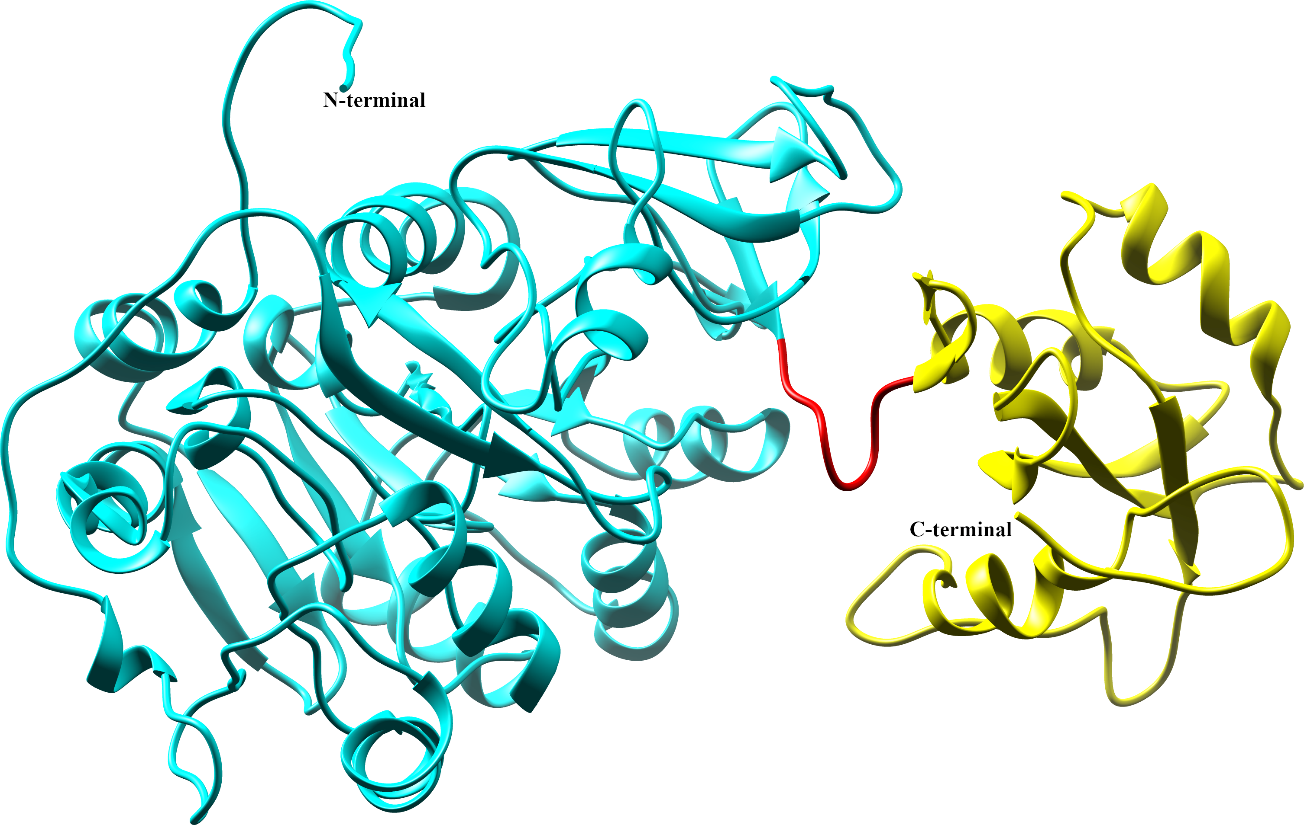


**Supplementary Fig. 3**. Predicted 3D structure of PqsA. The N-terminal of the protein is shown in cyan color, the C-terminal is shown in yellow, and the small hinge region responsible for connecting the N- and C-terminal domain is shown in red.


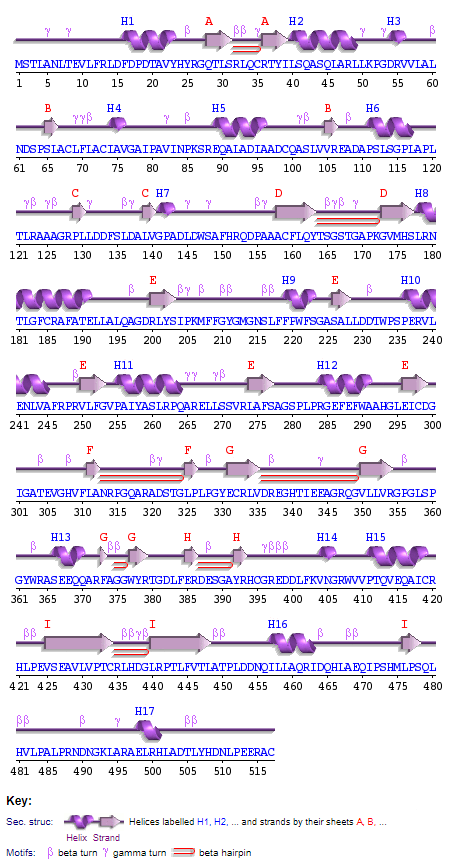


**Supplementary Fig. 4.** Secondary structure of the modeled PqsA enzyme.


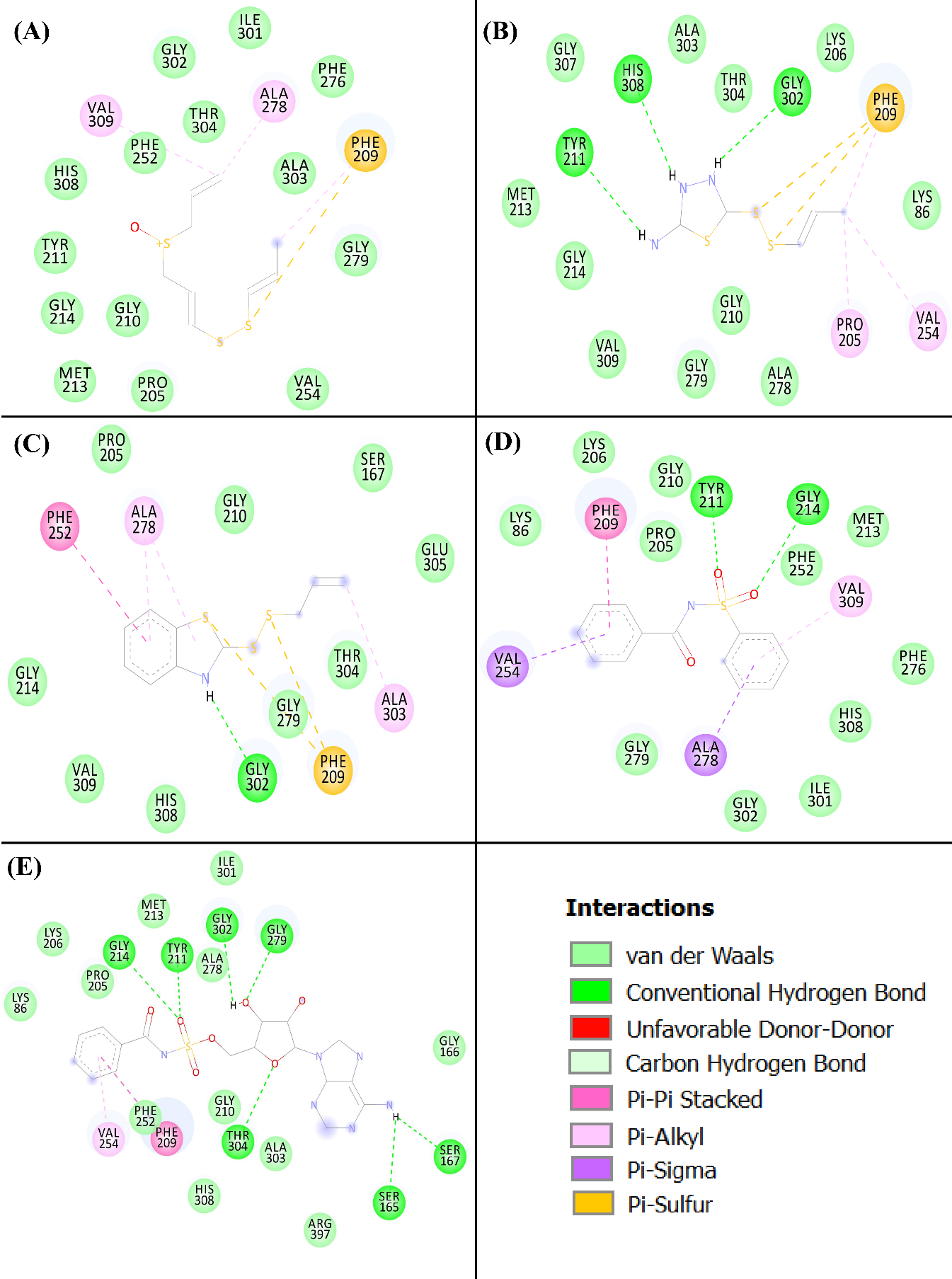


**Supplementary Fig. 5.** 2D interaction between the enzyme and known inhibitors. (A)–(E) represent compounds 1–5, respectively.

**Binding interactions and interacting residues between enzyme and known compounds**

Major interactions found in compound 1 are two alkyl and pi-alkyl interactions with the pocket residues Val309 and Ala278. two pi-sulfur bonds with Phe209. The remaining key residues His308, Phe252, Thr304, Gly302, Ile301, Phe276, Ala303, Gly279, Val254, Pro205, Met213, Gly210, Gly214, and Tyr211 are involved in van der Waals interactions. The reported experimental IC_50_ value of this compound is 15 ± 2.64 *µ*M and showing *in-silico* binding affinity of −4.4 kcal/mol.

Compound 2 is able to make eight interactions with the residues in the PqsA active pocket. There are three conventional hydrogen bonds with His308, Tyr211, and Gly302. Three pi-alkyl interactions with Phe209, Val254, and Pro205 and three pi-sulfur bonds with Phe209. Moreover, the residues Gly307, Ala303, Thr304, Lys206, Lys86, Ala278, Gly210, Gly279, Val309, Gly214, and Met213 are involved in van der Waals interactions. The IC50 value of this compound was reported as 2.28 ± 0.11 *µ*M and the predicted binding affinity is −5.0 kcal/mol.

Compound 3 displays seven interactions: three pi-alkyl interactions with Ala278 and Ala303, two pi-sulfur interactions with Phe209, one hydrogen bond with Gly302, one pi−pi stacked interaction with Phe252. The residues Pro205, Gly210, Ser167, Glu305, thr304, Gly279, His308, Val309, and Gly214 are involved in van der Waals interactions. This reported compound has an IC50 value of 0.98 ± 0.14 *µ*M and the predicted binding affinity with enzyme is −5.6 kcal/mol.

Compound 4 has six bonds: two pi-alkyl interactions with Phe209 and Val309, two conventional hydrogen bonds with Tyr211 and Gly214, and two pi-sigma interactions with Val254 and Ala278. Moreover, the residues Lys86, Lys206, Pro205, Gly210, Phe252, Met213, Phe276, His308, Ile301, Gly302, and Gly279 are involved in van der Waals interactions with the compound. The IC50 value of this compound is 36.2 ± 2.39 *µ*M and the predicted binding affinity is −7.6 kcal/mol.

Compound 5 displays more stable binding with PqsA by allowing seven conventional hydrogen bonds to interact with the most conserved residues Gly214, Tyr211, Gly302, Gly279, Ser167, Ser165, and Thr304, one pi-alkyl interaction with Val254 and one pi-pi stack interaction with Phe209. The residues Lys86, Lys206, Pro205, Met213, Ala278, Ile301, Gly166, Arg397, Ala303, His308, and Phe252 are involved in van der Waals interactions. This active compound is reported with its constant Ki value used to represent inhibitory potency of 16.5 ± 2.6 nM and the predicted binding affinity is −8.6 kcal/mol.

**Supplementary Table 1.** Top 10 predicted compounds from analogue library and their binding affinity and interacting residues. Compounds 826, 812, and 611 are the analogues of compound 4, while the rest of the compounds are the analogues of compound 5.

| **Compounds** | **Interacting residues** | **Binding affinity (kcal/mol)** |
| --- | --- | --- |
| 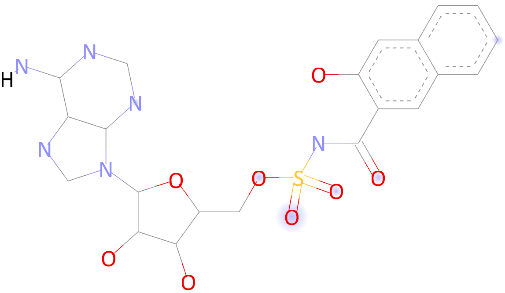  Compound 1084 | Pro205, Phe252, Met213, Gly214, Phe209, Ala278, Gly279, Tyr211, Gly210, Ser280, Val309, His308, Thr304, Ser165, Gly302, Ala303, Thr164, His394, Ser167, Glu305, Lys172, Thr168, Arg397, Asp382, Thr380 | −9.3 |
| 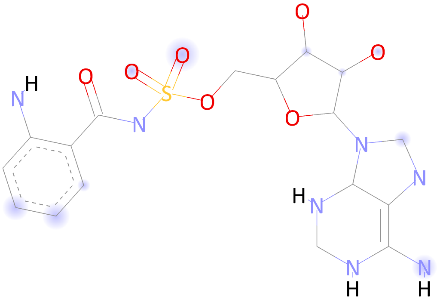  Compound 1066 | Pro205, Met213, Phe209, Gly214, Phe252, Gly210, Tyr211, Ala278, Thr304, His308, Gly279, Ser280, Val309, Ala303, Ser167, Glt307, Glu305, Lys172, Gly302, Thr168, Tyr362, His394, Arg397, Asp382, ly381, Gly381, Thr380, Tyr378, Arg379 | −9.1 |
| 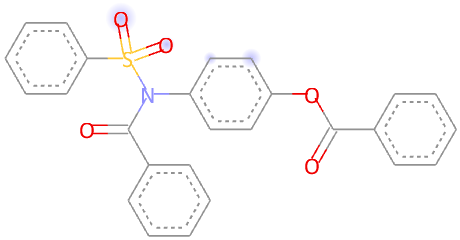  Compound 826 | Val254, Gly253, Phe252, Pro205, Met213, Gl214, Phe209, Gly212, Ala278, Ser280, Gly279, Gly210, Tyr211, Ile301, Gly302, Thr304, Ala303, Phe310, Ser167, Glu305, His394, Arg397, Asp382, Thr380 | −8.9 |
| 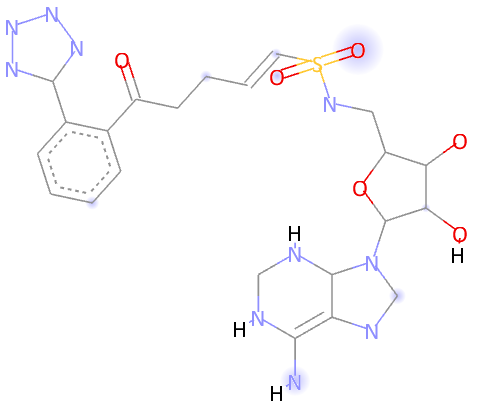  Compound 1527 | Pro205 Gly214, Met213, Tyr211, Phe209, Phe252, Gly253, Gly210, His308, Val254, Val309, Thr304, Thr164, Ala278, Gly300, Gly302, Ala303, Glu305, Tyr362, Lys172, Gly279, Ile301, Ser280, Ser167, Tyr378, Thr168, Thr380, Gly381, His394, Asp382, Arg397 | −8.8 |
| 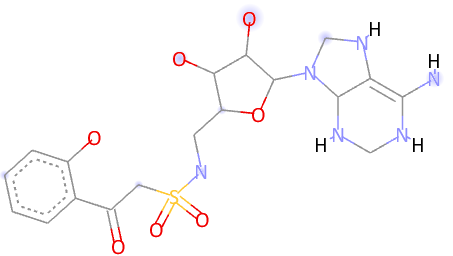  Compound 1237 | Lys206, Pro205, Phe252, Met213, Gly214, Val254, Gly212, Ala278, Phe209, Tyr211, Gly210, Gly279, Val309, His308, Ser380, Pro381, Gly302, Thr304, Ala303, Thr164, Glu305, His394, Ser167, Lys172, Arg397, Asp382, Thr380, Gly381, Tyr378, Thr168, Lys172, Tyr362 | −8.8 |
| 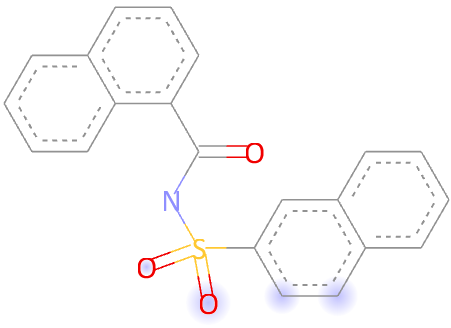  Compound 611 | Lys206, Pro205, Met213, Gly214, Phe252, Phe209, Tyr211, Gly210, Ala278, His308, Thr304, Gly279, Val309, Ser380, Gly302, Ala303, Ile301, Phe310, His394, Arg397, Asp382 | −8.6 |
| 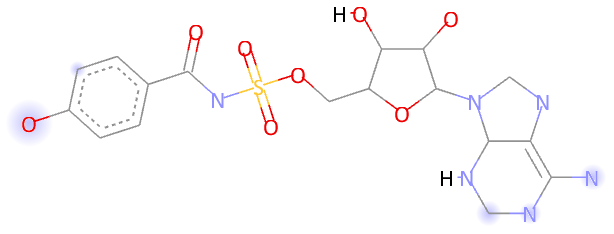  Compound 1397 | Lys206, Pro205, Val254, Phe252, Met213, Phe209, Gly214, Gly212, Asn215, Gly210, Ser165, Tyr211, Ala278, Ser280, Gly279, Gly300, Thr304, Val309, His308, Ser167, Ala303, Gly302, Ile301, Arg397, Asp382 | −8.6 |
| 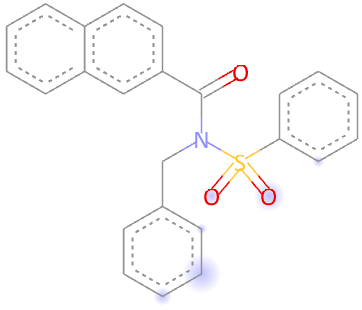  Compound 812 | Pro205, Phe252, Met213, Gly214, Phe209, Tyr211, Gly210, Ala278, Gly279, His308, Val309, Thr304, Ser165, Ser380, Ile301, Gly302, Ala303, Phe310, Ser167, His394, Tyr392, Arg397, Asp382 | −8.5 |
| 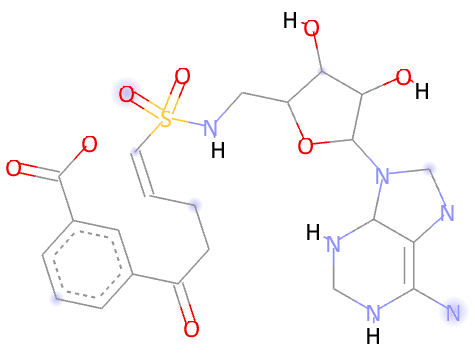  Compound 1223 | Pro205, Gly253, Phe252, Met213, Gly214, Val254, Phe209, Ala278, Gly210, Tyr211, Gly279, Ser380, His308, Thr304, Pro281, Val309, Gly302, Ala303, Ser167, Gly307, Glu305, Lys172, Thr168, Tyr362, His364, Arg397 | −8.3 |
| 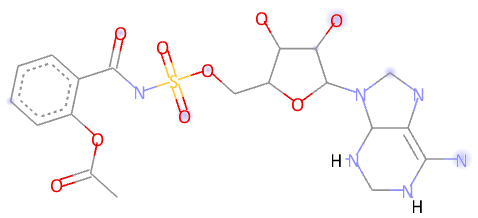  Compound 1061 | Pro205, Phe252, Met213, Gly214, Phe209, Phe276, Tyr211, Gly210, Ala278, Gly279, Ser280, Val309, His308, Thr304, Gly300, Pro381, Ile301, Gly302, Ala303, Glu305, Ser167, Lys172, His394, Arg397, Thr168, Tyr362, Asp382, Thr380, Tyr378 | −8.2 |
